# A phosphorylation map of the bovine papillomavirus E1 helicase

**DOI:** 10.1186/1743-422X-3-13

**Published:** 2006-03-08

**Authors:** Michael R Lentz, Stanley M Stevens, Joshua Raynes, Nancy Elkhoury

**Affiliations:** 1 Department of Biology, University of North Florida, 4567 St. Johns Bluff Rd., S., Jacksonville, FL 32224, USA; 2 Proteomics Core, Interdisciplinary Center for Biotechnology Research, University of Florida, Gainesville, FL 32610, USA

## Abstract

**Background:**

Papillomaviruses undergo a complex life cycle requiring regulated DNA replication. The papillomavirus E1 helicase is essential for viral DNA replication and plays a key role in controlling viral genome copy number. The E1 helicase is regulated at least in part by protein phosphorylation, however no systematic approach to phosphate site mapping has been attempted. We have utilized mass spectrometry of purified bovine papillomavirus E1 protein to identify and characterize new sites of phosphorylation.

**Results:**

Mass spectrometry and *in silico *sequence analysis were used to identify phosphate sites on the BPV E1 protein and kinases that may recognize these sites. Five new and two previously known phosphorylation sites were identified. A phosphate site map was created and used to develop a general model for the role of phosphorylation in E1 function.

**Conclusion:**

Mass spectrometric analysis identified seven phosphorylated amino acids on the BPV E1 protein. Taken with three previously identified sites, there are at least ten phosphoamino acids on BPV E1. A number of kinases were identified by sequence analysis that could potentially phosphorylate E1 at the identified positions. Several of these kinases have known roles in regulating cell cycle progression. A BPV E1 phosphate map and a discussion of the possible role of phosphorylation in E1 function are presented.

## Background

Papillomaviruses infect epithelial cells of cutaneous or mucosal origin in a variety of vertebrate hosts. An infection is established in the basal layer of the epithelium, and a complex viral life cycle is carried out, dependent on the differentiation state of the host cell [1-3]. Upon entry into a basal epithelial cell, the infecting genome is transiently amplified to approximately 50 to 200 copies, establishing a latent infection. As latently infected cells divide, the viral genomes are replicated on average once per cell cycle to maintain this low genome copy number [4,5]. Minimal viral gene expression is observed during the latent period. As progeny cells migrate towards the epithelial surface, a differentiation pathway is triggered, leading to changes in viral gene expression, genome amplification, and assembly of progeny virions.

The papillomavirus genome must undergo three distinct modes of DNA replication during the course of an infection: transient amplification immediately upon infection; regulated replication during latency to maintain a constant copy number; and vegetative replication to amplify copy number prior to virion assembly. Viral DNA replication is initiated by the E1 protein, a virally-encoded nuclear phosphoprotein [6]. Along with the viral E2 protein, E1 identifies and binds the viral origin DNA sequence, distorts and unwinds the parental double helix, and recruits the host cell replication machinery by direct interactions with host replication proteins [7-9]. E1 is an ATP-dependent DNA helicase that unwinds DNA at the viral replication fork, while other replication functions are supplied by the host cell (reviewed in [10]).

We and others have proposed that the complicated regulation observed for papillomavirus DNA replication is imposed by host cell regulatory mechanisms [11-16]. Cell cycle progression and cellular differentiation are controlled in part by phosphorylation of key target proteins by cellular kinases (reviewed in [17-20]). Several labs are investigating the role of E1 phosphorylation on bovine papillomavirus DNA replication activity, and have provided strong evidence that viral DNA replication is regulated by E1 phosphorylation [11,12,21-23]. A number of individual phosphorylation sites on BPV E1 have been identified by several groups, but no systematic effort to identify all of the phosphorylated amino acid positions of this protein has been undertaken. Here we report five previously unidentified phosphorylation sites and confirm two known sites, identified by a combination of mass spectrometry (MS) methods. With sites previously identified by other methods, this brings the total number of phosphate positions on BPV E1 to ten. This E1 phosphate map will provide a new tool to more fully understand viral replication and serve as a useful model for investigating regulation of viral and cellular DNA replication.

## Results

### Identification of E1 phosphopeptides by mass spectrometry

**Figure 1:**
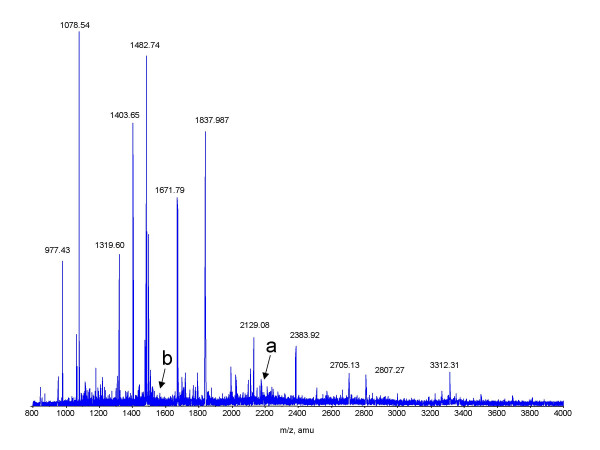
**MALDI-qTOF MS analysis of E1 tryptic digest**. Protein characterization by peptide mass fingerprinting allowed for over 50% sequence coverage of the E1 phosphoprotein. Letters "a" and "b" indicate two peaks of low signal intensity corresponding to the phosphopeptides LDLIDEEEDpSEEDGDSMR and VLpTPLQVQGEGEGR, respectively.

Very little E1 protein is produced during the course of an infection or in BPV transformed cells. In order to generate quantities of purified E1 necessary for mass spectrometry analysis, E1 protein was isolated and purified from insect Sf9 cells infected with a recombinant baculovirus [23-25]. Samples of purified E1 were separated from protein contaminants by electrophoresis through polyacrylamide gels, stained with coomassie brilliant blue, and the E1 band cut from the gel. Protease digestions were performed directly in the gel slice. Phosphopeptides generally exhibit low ionization efficiencies which makes mass detection difficult. Furthermore, stoichiometry of phosphorylation can be relatively low, further complicating detection. A combination of mass spectrometric-based methods was therefore used to identify major phosphorylation sites on the E1 protein. Matrix-assisted laser desorption/ionization (MALDI) and electrospray ionization (ESI) quadrupole time-of-flight (qTOF) mass spectrometry were employed since both ionization techniques have been shown to provide complementary information from peptide mass fingerprint (PMF) analysis [26,27]. Coupling HPLC to ESI also increases observation of phosphopeptides by minimizing signal suppression from other more abundant peptides. Fig. 1 is a MALDI-qTOF mass spectrum displaying the tryptic fragment profile obtained after in-gel digestion of the E1 phosphoprotein. From this analysis, low-abundance peptides could be observed with enough signal intensity in order to sequence and potentially identify sites of phosphorylation. For example, tryptic peptides containing the phosphorylated residues S584 and T126 (m/z 2176 and 1562, respectively, identified on Fig. 1) were present at low abundance, however, enough sequence ions were produced upon collision-induced dissociation (CID) to determine that the peptides were phosphorylated (data not shown). These and other peptides were singled out for further analysis because their mass corresponded to that of a potentially phosphorylated peptide.

**Figure 2:**
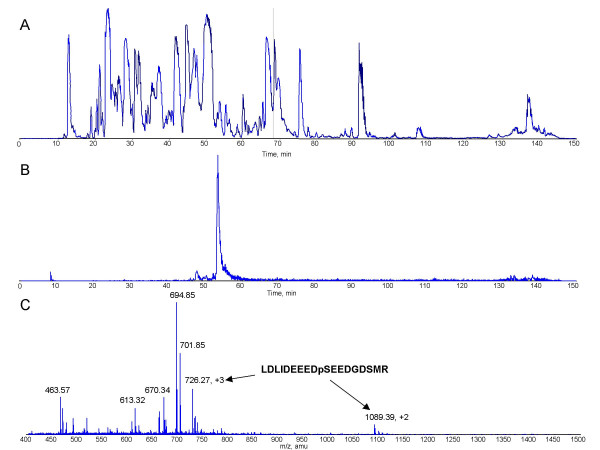
**Tandem mass spectrometric analysis of the E1 tryptic digest**. A. Base peak ion chromatogram obtained by HPLC/ESI-qTOF MS and MS/MS analysis of the E1 tryptic digest. B. Selected ion retrieval for *m/z *1088.8, which corresponds to the doubly-charged tryptic phosphopeptide LDLIDEEEDpSEEDGDSMR. C. Full scan mass spectrum at RT 53.1 min showing the presence of several tryptic peptides including the doubly and triply-charged phosphopeptide LDLIDEEEDpSEEDGDSMR.

Since the whole tryptic digest had been placed on a single MALDI target spot for the PMF analysis, signal suppression of other components within the mixture including phosphorylated peptides can occur. For this reason, ESI was utilized given the feasibility of coupling liquid chromatographic techniques to this particular ionization source. Fig. 2 demonstrates a tandem mass spectrometric analysis of the E1 tryptic digest. Fig. 2A is the base-peak ion chromatogram obtained upon rpHPLC-qTOFMS and MS/MS analysis of the E1 tryptic digest mixture. The complexity of the digest mixture is apparent from the ion chromatogram, demonstrating the advantage of HPLC separation prior to mass spectrometric analysis in terms of minimizing signal suppression. The peptide with *m/z *1088.8 is predicted to correspond to the tryptic peptide containing phosphoserine 584. Selected ion retrieval was performed, and the data shown in fig. 2B. The full-scan mass spectrum of this peak (Fig. 2C) reveals several peptides corresponding to the doubly- and triply-charged tryptic peptide in which serine 584 is phosphorylated.

**Figure 3:**
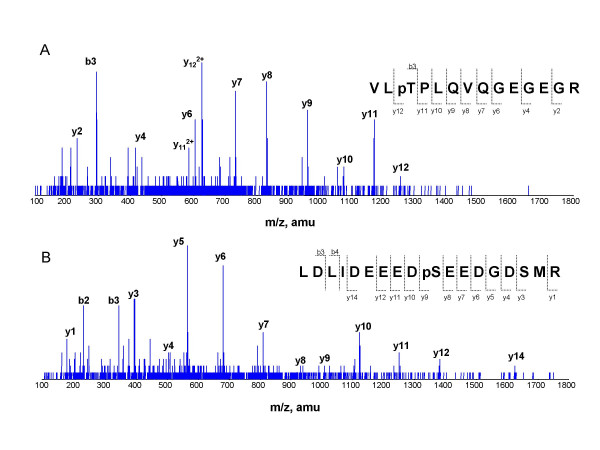
**MS/MS spectra of the phosphopeptides VLpTPLQVQGEGEGR and LDLIDEEEDpSEEDGDSMR**. Low-energy sequence ions (b and y-type ions) produced by collision-induced dissociation allowed for identification of several E1 phosphorylation sites after searching the tandem MS data against the NCBI nr sequence database with the MASCOT algorithm. A. Spectrum for VLpTPLQVQGEGEGR. B. Spectrum for LDLIDEEEDpSEEDGDSMR. The b and y-type ions are indicated on the peptide sequence and on the corresponding spectrum peak. A differential modification of 80 Da for serine and threonine was included in the MASCOT search parameters.

After searching the tandem mass spectrometric data with the Mascot database search algorithm, several sites of phosphorylation including those observed from MALDI analysis were identified. Fig. 3 displays MS/MS spectra of the phosphopeptides LDLIDEEEDpSEEDGDSMR and VLpTPLQVQGEGEGR obtained from tandem mass spectrometric analysis of the E1 tryptic digest. This data is representative of data collected for other identified phosphopeptides. "y" peaks correspond to ion fragments derived from the carboxyl terminus of the tryptic peptide, and "b" ions are generated from the amino terminal end. In general, the low-energy fragment ions observed for each MS/MS spectra covered enough of each peptide sequence to identify the residue in which phosphorylation had occurred. A list of the total phosphorylation sites identified by mass spectrometric analysis is provided in table 1.

### *In silico *sequence analysis of BPV E1 protein

Unknown phosphorylation sites on proteins can be predicted by newly developed algorithms. These programs use neural networks to predict unknown phosphorylation sites based on the sequence context of known sites in phosphoproteins. The BPV E1 protein sequence was submitted to NetPhos 2.0 [28], and the results are included in table 1. All of the sites identified in this study are predicted by this program, however some are predicted only weakly. There are nineteen other predicted serine or threonine sites that have not been positively identified (data not shown; see discussion), as well as seven tyrosines. It has previously been shown that BPV E1 labelled in vivo with ^32^P phosphate does not contain label on tyrosine, as shown by phosphoamino acid analysis [21,24]; therefore the predicted tyrosine sites will not be further considered. In order to identify the kinases most likely to target the sites we identified, the BPV E1 sequence was analyzed by manual sequence analysis and through NetPhosK 1.0, which predicts the most probable kinases based on information from evolutionarily conserved sites on known phosphoproteins [29]. The cellular kinases predicted to modify the phosphorylation sites were determined and included in table 1. The complete list includes ATM, CDK, CK1, CK2, DNAPK, p38MAPK, and RSK, however it is possible that not all of these predicted kinases interact with E1. Several sites are potential targets of multiple kinases with similar probabilities. Determining the relevant kinases for E1 phosphorylation in a complete infection cycle in the natural host is not possible at this time.

## Discussion

**Figure 4:**
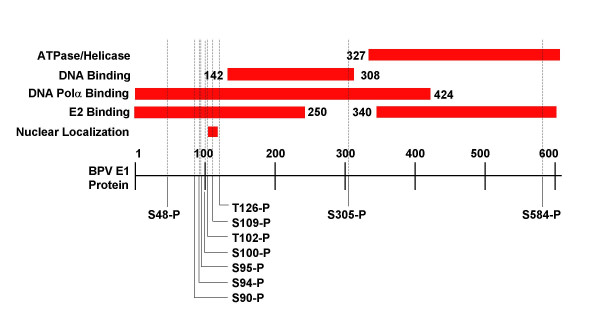
**Positional phosphate map and functional domains of the BPV E1 protein**. The 605 amino acid protein is represented on the lower horizontal line. The position of each of the phosphorylated amino acids is shown below. Functional domains are represented by the solid bars above. Functional domain boundaries in most cases were determined by deletion mutagenesis analysis as described in [10] and references therein.

Using MS analysis, we have identified five new phosphoamino acid positions on insect cell derived BPV E1 protein, and confirmed two others previously identified through mutation analysis. Taken with other previously published sites (threonine 102 [21], serine 90 [30], and serine 109 [23]) we present a map of the major sites of phosphate addition on this viral DNA helicase. This map is shown in fig. 4. In total there are ten sites: serines 48, 90, 94, 95, 100, 109, 303, and 584; and threonines 102 and 126. This data correlates well with previously published *in vivo *labelling and phosphoamino acid analysis data, in which phosphoserine accounts for approximately 90 percent of the label, with phosphothreonine contributing the remaining ten percent [21,24].

This and previous studies to identify *in vivo *E1 phosphate sites have been carried out on protein derived from baculovirus infected insect cells. We acknowledge the potential for variation from this cell line and the natural mammalian host, however, there is currently no system in which sufficient quantities of E1 protein can be generated from mammalian cells for these mapping studies. When direct comparison has been possible, it is observed that protein phosphorylation patterns in mammalian and insect systems are very similar, varying primarily quantitatively rather than qualitatively [31]. We are confident that the sites described here are comparable to the map that would be derived from E1 protein produced in mammalian cells. A direct comparison is desirable, and efforts will continue to develop a system for high-level E1 expression in a mammalian cell line.

The map presented here does not take into account any differences in the proportion of the protein sample that has phosphate at a particular site versus those that do not. We expect that phosphorylation/dephosphorylation will vary with the cell cycle and/or through the viral life cycle. Some sites may be only transiently phosphorylated, or phosphorylated only in more differentiated cells, and may therefore be missed in this screen. Late stage baculovirus infected cells are predominantly in the G2 stage of the cell cycle [32]. The phosphorylation pattern of our protein sample may therefore vary either quantitatively or qualitatively from protein found in natural host cells infected with BPV. Phosphate site analysis of E1 prepared from different cell cycle stages of synchronized cells would be highly desirable, but is not possible at this time.

NetPhos2.0 predicts phosphorylation of 17 serines or threonines that have not been identified as phosphate sites. This is not surprising since the algorithm used characterizes the local amino acid sequence only, and does not take into account three-dimensional structure, subcellular localization, or other structural features [28]. By ignoring these important structural and functional features, prediction algorithms identify sites that may be unrealistic in the cellular setting. Nevertheless, it is possible that our analysis has missed one or more rare or transient sites.

Using the kinase prediction program NetPhosK, the list of potential kinases targeting the known E1 phosphate sites is large, including ATM, CDK, CK1, CK2, DNAPK, p38MAPK, PKA, PKC, PKG, and RSK. It is unlikely that all of the predicted enzymes interact with E1. The prediction algorithm compares the submitted amino acid sequence to known sites [29]. It does not take into account cell type, subcellular localization, protein function, or other potentially important features, however it defines a useful starting point for further analysis. Based on E1's role in viral DNA replication, kinases known to be involved in cell cycle progression or DNA metabolism seem most likely to be involved in E1 modification. Five of the identified serines are in a consensus for the kinase CK2, and two others are likely cyclin/Cdk sites. Two are predicted targets for protein kinase C (PKC), and one by DNA-dependent protein kinase (DNAPK). Serines 90, 109, and 584 (CK2); and threonine 102 (cyclin Cdk) were previously shown to be phosphorylated by the predicted kinases *in vitro *[21,23,24,30]. Serines 90 and 109 are in consensus sequences for PKC. These sites were previously shown to be phosphorylated by mutation analysis, but were not identified in this MS screen. It is possible that the relevant PKC isozymes(s) are less active in late stage baculovirus infected insect cells. This enzyme consists of a family of at least twelve isozymes implicated is a wide variety of cell signalling pathways, including the G1/S cell cycle transition [33,34]. Other known functions include a role in regulating differentiation of epithelial tissue, and could therefore couple viral DNA replication to the differentiation state of the host cell [23,35-40].

CK2 is predicted to phosphorylate half of the sites identified on the E1 protein. CK2 is a ubiquitous enzyme whose role in the cell is under investigation but is still poorly defined. A wide range of identified CK2 substrates implicates this kinase in a number of critical cell functions, including cell cycle regulation, cell survival, and regulation of gene expression [18,20,41-44]. A substantial proportion of known CK2 substrates are viral in origin [44]. In previous experiments, two CK2 sites on BPV E1 (serines 48 and 584) were studied by mutation analysis. Mutation of either site to alanine completely eliminated viral replication, while acidic substitutions restored replication function [12,45]. The specific role of these and other CK2 sites in E1 function remains to be determined.

BPV DNA replication has been shown to require binding of E1 by cyclin E-Cdk2 in a *Xenopus *extract system, although the specific role of the cyclin/kinase was not determined [11]. Phosphorylation of HPV-11 E1 by Cdk was recently shown to regulate nuclear entry of the protein by masking a nuclear export signal, however this signal is not contained in the BPV E1 sequence [46,47]. There are three potential sites for Cdk phosphorylation in BPV E1 (threonines 102 and 126; serine 283); the specific amino acid(s) required for replication in the *Xenopus *system were not identified. Point mutations at threonine 102 or serine 283 do not significantly alter DNA replication in a transient system [[21]; Lentz, unpublished results], and serine 283 has not been shown to be a target for phosphorylation in this or other studies. A recent model proposes that BPV E1 concentration controls viral DNA replication in latently infected cells [11,13,14]. In this model, E1 is targeted for degradation by the anaphase promoting complex, and is stabilized at the G1/S transition by interaction with cyclin E/Cdk2. Our data supports this model by identifying threonine 126 as a potential target of the Cdk activity. A functional analysis of threonine 126 may clarify the role of Cdk in BPV DNA replication.

Of the ten phosphate sites, six are tightly clustered within 20 amino acids, between serines 90 and 109. Two more lie on either side of this cluster, at position 48 and 126. This clustering is easily seen in fig. 4. It is notable that the majority of the phosphate sites are concentrated on the amino-terminal domain of the protein. This region of the protein is the least conserved among the many E1 protein sequences that have been determined to date, and has few common functions among different E1 proteins [10]. The only conserved functional domain identified in this region of the protein is the nuclear localization signal. This and other BPV E1 functional domains are identified in fig. 4. In both BPV-1 E1 and HPV-11 E1, the carboxyl-terminal two-thirds can function in replication following truncation of the amino-terminus, suggesting only a supporting or regulatory role for the amino terminal domain [48-50]. In BPV E1, known functions of the amino terminal domain include nuclear import, and interaction domains for the viral E2 protein and cellular DNA polymerase alpha [[10] and references therein]. More recently, several crystal structures implicate the carboxyl-terminal domain in several key E1 functions including E1 dimerization, E1-DNA interactions, and E1–E2 interactions. These structures include an origin assembly intermediate between the helicase catalytic domain of HPV-18 E1 and the viral E2 protein [51]; a dimer of BPV E1 DNA binding domains [52]; and the BPV E1 DNA binding domain bound to origin DNA [53]. These structures demonstrate that the amino-terminal region of E1 is not required for these protein-protein or protein-DNA interactions. The essential DNA binding, ATPase, and helicase domains are all located in the carboxyl-terminal domain where only two phosphate sites, serines 305 and 584, are located [10]. Serine 305 is located in the DNA binding domain, but is not in a readily identifiable kinase motif, and is poorly conserved among E1 proteins. Serine 584 is near the end of the consensus D box of the ATPase domain. Mutation of serine 584 to alanine abolishes DNA replication in a transient assay, however bacterially derived E1 protein functions as a helicase *in vitro*, so the precise role of this phosphorylation event remains unclear [12]. Our map supports a general model in which the amino terminal domain of BPV-1 E1 regulates or enhances E1 function, while the carboxyl domain provides essential DNA binding and enzymatic activities. We hypothesize that phosphorylation within the amino terminal domain contributes to regulation or enhancement of E1 function. Our phosphate site map will be useful for directing future molecular analysis of the role of phosphorylation in E1 function and viral DNA replication.

## Conclusion

This report describes an analysis of phosphorylation sites of the BPV E1 helicase by mass spectrometry methods. Five previously unknown sites were identified, and two previously known sites were confirmed. Taken with other known sites, there are at least ten amino acids on E1 that are phosphorylated. The position of the phosphate sites on the protein and the kinases predicted to interact with E1 support a model in which phosphorylation of E1 enhances or regulates its activities during the complex viral life cycle.

## Methods

### Protein expression and purification

E1 protein was synthesized in and purified from recombinant baculovirus infected insect cells as previously described, with several modifications [23]. Briefly, *Spodoptera frugiperda *Sf9 cells were maintained as adherent cultures in TNMFH medium supplemented with 10% (v/v) fetal bovine serum (JRH Biosciences), penicillin, and streptomycin. Generation of recombinant baculovirus expressing FLAG-tagged BPV E1 protein under control of the polyhedrin promoter was described previously [23]. Protein was prepared from ten, 10 cm dishes of adherent Sf9 cells 48 hours post-infection (pi). 30 minutes prior to harvest, cells were treated with 10 nM calyculin A, a PP1 and PP2A phosphatase inhibitor. Adherent cells were scraped into the culture medium, and along with any detached cells were pelleted and stored at -80°C.

Protein was extracted from salt-washed nuclei as described [25]. E1 purification was carried out by passing extracts over a column containing M2 anti-FLAG antibody bound to sepharose beads (Sigma). After washing to remove unbound proteins, the FLAG-E1 protein was eluted with synthetic FLAG peptide according to the manufacturers directions (Sigma). Fractions were analyzed by polyacrylamide gel electrophoresis and E1 containing fractions were pooled and concentrated by dialysis against solid sucrose, followed by dialysis into E1 storage buffer (50 mM Tris-HCl (pH 7.8), 1 mM EDTA, 1 mM dithiothreitol (DTT), 12.5 mM MgCl_2_, 100 mM KCl, 0.3 mM NaCl, 10% (v/v) glycerol). Purified E1 protein was electrophoresed into 10% SDS polyacrylamide gels and stained with coomassie brilliant blue. The E1 containing gel fragments were isolated, digested in-gel with trypsin, and the corresponding tryptic peptides were used directly for mass spectrometry analysis.

### Mass spectrometry analysis of purified E1 protein

Capillary reversed-phase (rp) HPLC separation of E1 protein digests was performed on a 15 cm × 75 μm i.d. PepMap C18 column (LC Packings, San Francisco, CA) in combination with an Ultimate Capillary HPLC System (LC Packings, San Francisco, CA) operated at a flow rate of 200 nL/min. A capillary trap with the same stationary phase chemistry as the analytical column was used in combination with the Switchos isocratic solvent delivery pump in order to concentrate and desalt the sample prior to LC/MS/MS analysis. Gradient flow rates between 200–300 nl/min were obtained by splitting a flow of 180 μL/min supplied by the gradient HPLC pump. Solvent A was 0.1% acetic acid in 95% water / 5% acetonitrile and solvent B was 0.1% acetic acid in 10% water / 90% acetonitrile. Following isocratic solvent delivery for 5 minutes during the sample desalting step, a linear gradient was carried out for 120 min to 40% solvent B. Inline mass spectrometric analysis of the column eluate was accomplished by a hybrid quadrupole time-of-flight instrument (QSTAR, Applied Biosystems, Foster City, CA) equipped with a nanoelectrospray source. The information-dependent acquisition (IDA) mode of operation was employed in which a survey scan from m/z 400–1500 was acquired followed by collision-induced dissociation (CID) of the two most intense ions. Survey and MS/MS spectra for each IDA cycle were accumulated for 1 and 3 sec, respectively.

Prior to MALDI-qTOFMS analysis, the digested samples were bound to a C18 ZipTip microcolumn, washed several times with 0.1% TFA, and eluted onto a MALDI target with 1 μL matrix solution. The matrix solution was prepared by dissolving 5 mg of a-cyano-4-hydroxycinnamic acid (Sigma-Aldrich, St. Louis, MO, USA) in 1 mL of 50% acetonitrile/0.1% TFA. Full scan mass spectra were acquired for 1 minute using a N_2 _laser operated at 20 Hz. For CID experiments in which MALDI was the source of ion production, collision energies were maintained between 75–110 eV using nitrogen as the collision gas.

Fragment ion data generated by the IDA and conventional MS/MS modes of acquisition via the QSTAR were searched against the NCBI nr sequence database using the Mascot (Matrix Science, Boston, MA) database search engine. Probability-based MOWSE scores above the default significant value were considered for peptide sequence identification in addition to validation by manual interpretation of the tandem MS data. Manual interpretation was also necessary for low-abundance and/or poorly-ionized phosphopeptides that did not demonstrate adequate MS/MS spectral quality for Mascot processing.

### *In silico *analysis

The full-length BPV E1 sequence was extracted from the Swiss-Prot sequence database, [Swiss-Prot:P03116] in FASTA format. The sequence was submitted online to NetPhos2.0 (, [28]) for analysis of potential phosphate sites, and to NetPhosK (, [29]) for identification of potential kinases that may interact with E1.

## Competing interests

The author(s) declare that they have no competing interests.

## Authors' contributions

MRL conceived of the study, directed the project, carried out the *in silico *analysis, and drafted the manuscript. SMS carried out all of the mass spectrometry and MS data analysis, and contributed to the draft of the manuscript. NE and JR generated the recombinant baculovirus, and expressed, purified, and analyzed protein samples. All authors read and approved the final manuscript.

## Figures and Tables

**Table 1:** E1 phosphate sites identified by MS analysis and site characteristics. Phospho-amino acid positions identified in this study are shown in the left column, followed by the sequence of the surrounding amino acids. The phosphorylated position is highlighted in bold type. The NetPhos2.0 and NetPhosK scores for each phospho-amino acid is shown. 1.0 is the maximum score, and 0.5 is the default threshold for likely phosphorylation. The kinases predicted by the NetPhosK algorithm are shown. Where there is no NetPhosK score, that position was not predicted and the kinases were identified by manual analysis and comparison to published consensus sequences [44]. *, phosphate sites identified by MS and not previously known; **, phosphate sites identified by MS, confirming previously known sites.

**Amino Acid**	**Sequence**	**NetPhos Score**	**Predicted Kinase**	**NetPhosK Score**
Ser 48**	VESDRYD**S**QDEDFVD	0.997	CK2 ATM DNAPK RSK	0.64 0.58 0.56 0.52
Ser 94*	VLGSSQN**S**SGSEASE	0.926	CK2	
Ser 95*	LGSSQNS**S**GSEASET	0.993	CK2	0.59
Ser 100*	NSSGSEA**S**ETPVKRR	0.333	CK1, CK2	
Thr 126*	NEANRVL**T**PLQVQGE	0.959	p38MPK	0.52
Ser 305*	AQTTLNE**S**LQTEKFD	0.253	DNAPK	0.53
Ser 584**	LIDEEED**S**EEDGDSM	0.998	CK2 CK1	0.69 0.53
